# Effects of land‐use change in the Amazon on precipitation are likely underestimated

**DOI:** 10.1111/gcb.15810

**Published:** 2021-08-17

**Authors:** Mara Baudena, Obbe A. Tuinenburg, Pendula A. Ferdinand, Arie Staal

**Affiliations:** ^1^ National Research Council of Italy Institute of Atmospheric Sciences and Climate (CNR‐ISAC) Torino Italy; ^2^ Copernicus Institute of Sustainable Development Utrecht University Utrecht The Netherlands

**Keywords:** column water vapor, deforestation, drought, moisture recycling, moisture tracking, rainfall, tropics

## Abstract

The Amazon forest enhances precipitation levels regionally as trees take up water from the soil and release it back into the atmosphere through transpiration. Therefore, land‐use changes in the Amazon affect precipitation patterns but to what extent remains unclear. Recent studies used hydrological and atmospheric models to estimate the contribution of tree transpiration to precipitation but assumed that precipitation decreases proportionally to the transpired portion of atmospheric moisture. Here, we relaxed this assumption by, first, relating observed hourly precipitation levels to atmospheric column water vapor in a relatively flat study area encompassing a large part of the Amazon. We found that the effect of column water vapor on hourly precipitation was strongly nonlinear, showing a steep increase in precipitation above a column water vapor content of around 60 mm. Next, we used published atmospheric trajectories of moisture from tree transpiration across the whole Amazon to estimate the transpiration component in column water vapor in our study area. Finally, we estimated precipitation reductions for column water vapor levels without this transpired moisture, given the nonlinear relationship we found. Although loss of tree transpiration from the Amazon causes a 13% drop in column water vapor, we found that it could result in a 55%–70% decrease in precipitation annually. Consequences of this nonlinearity might be twofold: although the effects of deforestation may be underestimated, it also implies that forest restoration may be more effective for precipitation enhancement than previously assumed.

## Graphical abstract

Forests in the Amazon affect precipitation via transpiration. We find that the relationship of atmospheric water vapor with observed precipitation events is strongly nonlinear. This implies that loss of atmospheric moisture due to forest loss has disproportionate effects on regional precipitation: A complete loss of the Amazon forest would lead to a 55%–70% decrease of the precipitation annually.

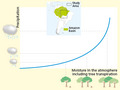

## 1 INTRODUCTION

Many tropical forests are currently undergoing land‐use changes, with forests being replaced by croplands, pastures, and other agriculture (Malhi et al., 2014). In the Amazon, deforestation is resurging after a decline during the early 21st century, and also fire occurrence is on the rise (Aragão et al., 2018; Barlow et al., 2020). This deforestation can affect precipitation levels: as trees photosynthesize, they contribute to evapotranspiration by pulling up water through their roots and releasing it back to the atmosphere through their leaves (Spracklen et al., 2018). In the southern Amazon, the dry season has been lengthening (Fu et al., 2013), and the number of dry days has been increasing (Espinoza et al., 2019). These changes have been related to modifications in large‐scale atmospheric circulation (Arias et al., 2015; Espinoza et al., 2019; Jiménez‐Muñoz et al., 2016; Leite‐Filho et al., 2020; Marengo & Espinoza, 2016). At the same time, it has been estimated that 20% of the annual precipitation across the basin has been transpired by trees (Staal et al., 2018): this mechanism is particularly important in the western Amazon and can mitigate droughts (Bagley et al., 2014; Espinoza et al., 2016; Mu et al., 2021), since the share of transpired moisture in precipitation increases as less atmospheric moisture is transported into the basin (Staal et al., 2018). Therefore, ongoing deforestation may intensify droughts (Bagley et al., 2014; Costa & Pires, 2010; Staal et al., 2020).

An effective method to map the effects of land‐use changes on precipitation levels is atmospheric moisture tracking. This technique uses atmospheric reanalysis data to simulate atmospheric moisture flows from evaporation to precipitation (Tuinenburg & Staal, 2020; Van der Ent et al., 2014). Detailed and realistic simulations of global moisture flows are possible for the recent past due to the availability of atmospheric reanalysis data (Tuinenburg et al., 2020). Together with spatially and temporally explicit models for the contributions of forest cover to evapotranspiration (Wang‐Erlandsson et al., 2014) or correlation‐based estimates of moisture enhancement by forests (Spracklen et al., 2012), the effects of hypothetical land‐use change on precipitation can be assessed.

Although atmospheric moisture tracking can provide important insights on land–atmosphere interactions, potential drawbacks may result from the fact that, by its very nature, the method reproduces actual past precipitation events. In other words, moisture tracking estimates moisture recycling (including that contributed by forest evapotranspiration) as it is, rather than how a reduction of evapotranspiration would have affected precipitation levels. The implicit assumption in these cases is that precipitation decreases in proportion to the loss of atmospheric moisture. However, both theory and observation indicate that this may be an oversimplification (Bretherton et al., 2004; Holloway & Neelin, 2009; Neelin et al., 2009). A strong nonlinear effect can occur above a threshold in atmospheric moisture (Peters & Neelin, 2006), which is generally attributed to the strongly nonlinear atmospheric dynamics, typically connected to convective precipitation (e.g., Baudena et al., 2008; D’Andrea et al., 2006; Peters & Neelin, 2006). Changes in atmospheric moisture content caused by land‐use changes might, thus, affect precipitation levels in yet unexplored ways. Here, we tested the null hypothesis that, on average, land‐use change affects precipitation proportionally to its effect on atmospheric moisture content. To test this “linearity assumption,” we determined the relations between atmospheric moisture content and precipitation rates based on empirical atmospheric reanalysis data for a large study area in the Amazon and surroundings. We selected a relatively flat area to avoid including the large effects of orography on (convective) precipitation. We coupled these relations to simulations tracking the moisture content of the atmosphere that has been transpired by trees across the whole Amazon basin. For the latter, we used the output from Staal et al. (2018), where monthly tree transpiration at 0.25° spatial resolution in the Amazon basin was estimated using a global hydrological model (Bosmans et al., 2017). The transpired moisture was subsequently tracked through the atmosphere using a detailed Lagrangian moisture tracking model (Tuinenburg et al., 2012). Taken together, this yielded monthly estimates of contributions of Amazon tree transpiration to regional precipitation levels at a 0.25° spatial resolution for the period 2003−2014 in the study area. This area has been estimated to depend strongly on moisture contributions from the Amazon (Staal et al., 2018; Zemp et al., 2014) and was, therefore, a particularly suitable case to test our hypothesis.

## 2 METHODS

### 2.1 Data description

**FIGURE 1:**
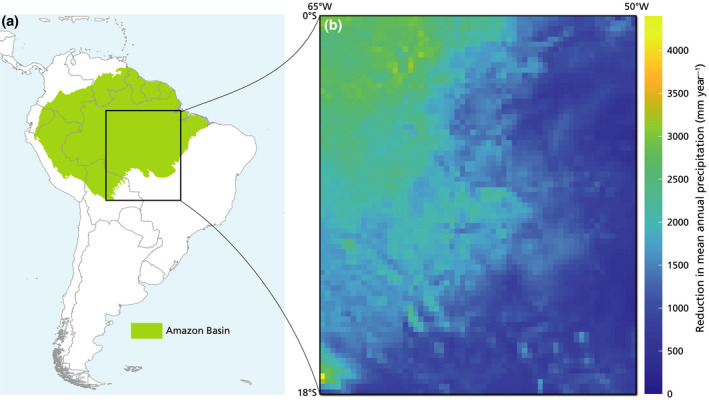
(a) Map of the Amazon (green‐shaded area), which is the source of the transpiration for the study area (black rectangle; across 0−18°S and 65−50°W). (b) Map of the average decrease of annual precipitation (mm year^−1^) in case of the absence of transpiration in the Amazon (calculated from the median values *p*_t,50_). The study period was 2003−2014

The study area covers a large, relatively flat (mean 227 m a.s.l., with a standard deviation of 166 m) part of the Amazon, between 0−18°S and 65−50°W (Figure 1), which includes the Amazon forest–Cerrado transition zone, containing forest, savanna, and agricultural areas. We excluded higher elevations within the Amazon basin to consider a rather homogeneous study area in terms of elevation; in this way, we minimized the known effects of orography on (convective) precipitation. The analyses were performed for the period 2003−2014 (following Staal et al., 2018) at a 0.25° resolution, resulting in 4320 spatial data points. We acquired hourly precipitation (*p*, mm h^−1^) and column water vapor (cwv, mm) from the ERA5 dataset for our study period and area (Hersbach et al., 2018). See the Supplementary material for the maps of the average annual precipitation (Figure S1) and column water vapor (Figure S2A), as well as the box plot of the distribution of cwv (Figure S2B) in the area.

We obtained the fraction (*f*_t_) of the column water vapor that was transpired from trees in the whole Amazon basin (Figure 1) from Staal et al. (2018), for each cell of 0.25° in our study area, and for each month during 2003−2014. First, that study estimates monthly transpiration throughout the Amazon, using the hydrological model PCR‐GLOBWB (Bosmans et al., 2017) to estimate forest contribution to evapotranspiration. Secondly, using a Lagrangian atmospheric moisture tracking model (Tuinenburg et al., 2012), Staal et al. (2018) simulate the subsequent atmospheric trajectories of the transpired moisture to evaluate the fraction of precipitation due to this transpired moisture. These fractions of precipitation from transpiration are assumed to be equivalent to the fraction of column water vapor from transpiration at the time of the precipitation event. Thus, we could use these fractions as estimates of *f*_t_. These estimates of transpiration recycling are robust against uncertainties in transpiration values, where transpired water column content increases slightly less‐than‐proportionally to the fraction of evapotranspiration that is attributed to transpiration (Staal et al., 2018). Finally, further tests performed by Staal et al. (2018) regarding a range of assumptions in atmospheric moisture tracking show that, although no major differences are expected under different assumptions, underestimation of moisture recycling is likely in areas and periods with large variability in vertical winds.

### 2.2 Analysis

First, we analyzed hourly precipitation as a function of total column water vapor for the study area. We fitted a linear and an exponential model to test the null hypothesis of linearity between these two variables. Furthermore, we calculated the median, first quartile, and third quartile (*p*_q_, with *q* = 50%, 25%, or 75%) of the distribution of the precipitation for different values of cwv, by binning the data along the cwv axis (every mm of cwv). For each bin, we calculated the *p*_q_ values only if there were more than five points in the bin, up to the maximum cwv retained value of 73 mm.

Next, for each grid cell across the study area, we estimated the column water content without the contribution from the Amazon (cwv_t_, mm) by multiplying the hourly ERA5 cwv time series by 1 − *f*_t_, where *f*_t_ are the estimates of the monthly contribution of tree transpiration from the Amazon by Staal et al. (2018). Finally, we used these estimated time series of atmospheric water content cwv_t_ to create three precipitation time series (*p*_t,q_, with *q* = 50%, 25%, or 75%) estimating the precipitation for each grid cell in the study area without the contribution of the Amazon. Specifically, we estimated *p*_t,q_ as a fraction *f*_q_ of the actual precipitation *p* from the ERA5 data set:

(1) $$p_{t,q}=f_{q}p,$$

where *f*_q_ is determined from the precipitation distribution quartiles *p*_q_. For each quartile *q*, *f*_q_ is the ratio between the precipitation values in the distribution quartiles corresponding to the column water vapor without the contribution of the Amazon (cwv_t_) and the column water vapor original value (cwv):

(2) $$f_{q}=\frac{p_{q}\left({\text{cwv}}_{t}\right)}{p_{q}\left(\text{cwv}\right)}.$$

In other words, for *q* = 25%, we calculated the ratio of the lower quartile precipitation values corresponding to cwv_t_ and cwv; the same procedure was repeated for the median and upper quartile. We then calculated precipitation daily and monthly time series across the area, for both the ERA5 data and the reduced precipitation *p*_t,q_. We used the daily results to assess the effects of land‐use change on precipitation events. Therefore, we removed from those results the days without precipitation (cut off at 0.01 mm day^−1^). We also considered longer‐term (monthly and annual) average precipitation reductions and compared them with the related cwv reductions.

To substantiate the analysis, we also implemented an alternative Monte Carlo–like approach, to estimate the same reduction of precipitation in the study area due to a lack of the contributions from transpiration in the Amazon basin. In this alternative approach, the time series of reduced column water vapor content cwv_t_ at an hourly time scale were produced in the same way as described above, but the reduced precipitation was calculated differently. Mainly, instead of using the quartile‐derived curves, we used a Monte Carlo procedure whereby for each value of hourly cwv_t_, a reduced precipitation value was randomly selected among all the precipitation values occurring for similar values of column water vapor (with replacement, i.e., we allowed the same precipitation value to be selected multiple times). The main advantage of this method was that it maintained the statistical distribution of the data. However, in contrast to the method described earlier, this procedure did not maintain the temporal structure within the data. For this reason, we analyzed only the total daily precipitation amount and the distribution of the daily precipitation for the data obtained with this approach.

## 3 RESULTS

**FIGURE 2:**
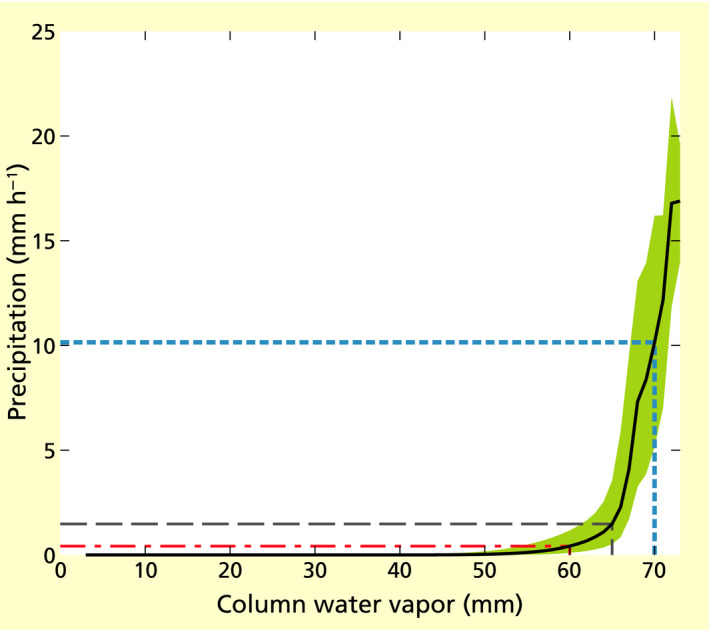
The relation between hourly precipitation (*p*, in mm h^−1^) and column water vapor (cwv, in mm) in the study area and period, according to ERA5 data. The black line gives the median (*p*_50_) and the shaded area the interquartile range (between *p*_25_ and *p*_75_). The relation is strongly nonlinear. To illustrate this, the black, long‐dashed line indicates that the precipitation at a cwv of 65 mm is equal to 1.5 mm h^−1^. At 5 mm lower cwv (red, dash‐dotted line), precipitation is 0.4 mm h^−1^, thus 1.1 mm h^−1^ lower than at cwv = 65 mm; at 5 mm h^−1^ higher cwv (blue, short‐dotted line), precipitation is 10.2 mm h^−1^, thus 8.7 mm h^−1^ higher than at cwv = 65 mm

We found that, in our study area, the relation between hourly precipitation and cwv was not well approximated by a linear relationship (*R* ^2^ = 0.07), while an exponential fit approximated it much better (*R* ^2^ = 0.29). In fact, the relationship was visibly strongly nonlinear (Figure 2): Hourly precipitation was negligible up to around 60 mm cwv, after which it rose sharply with a maximum relative increase (81%) between 66 and 67 mm cwv. Thus, the curve represented a superlinear increase in column water vapor with hourly precipitation. This is illustrated in Figure 2, where an increase in cwv from 65 mm to 70 mm is related to a much larger increase in hourly precipitation than an increase from 60 to 65 mm.

Based on the empirical (median) relation between precipitation and cwv in Figure 2, we considered the effects of the hypothetical removal of the contribution of the Amazon basin to transpiration on precipitation at different time scales. The observed median daily precipitation event size in the ERA5 data set for 2003−2014 in the study area was 4.30 mm day^−1^, which we estimated in the absence of tree transpiration from the Amazon basin would become 1.37 mm day^−1^, a decrease of median daily precipitation of 68%. We found comparable daily precipitation estimates for the alternative Monte Carlo–like approach, with a median of 1.41 mm day^−1^ per event, and thus a decrease of 67% (Figure S3).

Annually, on average, loss of transpiration decreased the estimated total precipitation by 70% (median) with an estimated range between 88% (*q* = 25%) and 61% (*q* = 75%) (Figure 2; Figure S3). This corresponded to an annual decrease of 1487 mm of precipitation (with 1866 mm for *q* = 25% and 1287 mm for *q* = 75%; Figure 1). For the alternative Monte Carlo approach, total precipitation across the 12 years was reduced by 55% with respect to the ERA5 data, corresponding to an average decrease of 1159 mm year^−1^ (Figure S3). For comparison, 13% of the column water vapor cwv for 2003−2014 in our study area (mean of the fraction values *f*_t_ from Staal et al. (2018)) originated from tree transpiration in the Amazon annually. See also Figure S4 for a map of the fraction of column water vapor in the study area that originates in the Amazon.

**FIGURE 3:**
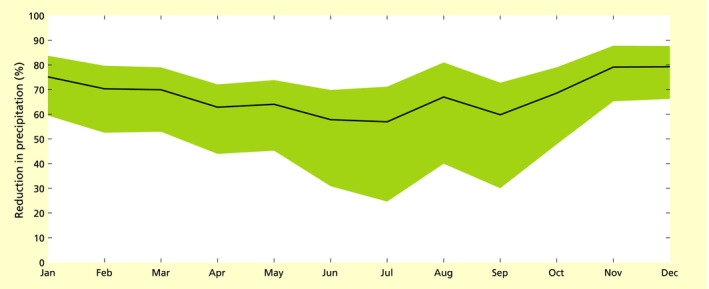
Monthly percentage reduction in precipitation in the study area for 2003−2014 due to the removal of the contribution of transpiration from the Amazon basin. The black line gives the percent reduction calculated from the median (*p*_t,50_) and the shaded area the interquartile range (calculated from *p*_t,25_ and *p*_t,75_)

The relative reduction in precipitation did not have a strong seasonal pattern (Figure 3). The absolute reduction followed the distribution of precipitation in the study area, with its highest value in January and the lowest value in July (Figure S5). Across all grid cells and months, median monthly precipitation reduction was 78%, with an estimated range between 100% (*q* = 25%) and 55% (*q* = 75%; Figure S6). For comparison, the reduction of column water vapor cwv displayed a more evident seasonal cycle (see Figure S7), similarly to what observed for the whole basin by Staal et al. (2018).

Geographically, there was no clear signal in the precipitation reduction along latitude (Figure 1; Figure S8). Instead, the precipitation reduction was most evident along the longitudinal direction (Figure 1; Figure S8), with a larger reduction in the western part of the study area (about 80% on average, Figures S8 and S9) than in the eastern part. This is expected, given the general east‐to‐west prevailing wind direction in the study area (Spracklen et al., 2012; Staal et al., 2018; Zemp, Schleussner, Barbosa, & Rammig, 2017).

## 4 DISCUSSION

We estimated that in a large and relatively flat study area, encompassing large part of the Amazon basin and including some surroundings in the south of it, the relationship between atmospheric moisture and precipitation was nonlinear: an average 13% reduction in atmospheric moisture due to transpiration loss (Staal et al., 2018) could potentially cause a 70% reduction in average precipitation annually (interquartile range: 61%−88%). This dramatic difference suggests that deforestation could have a much larger effect on precipitation in this region than previously thought. The estimated percentage was also larger than the Amazon's recycling ratio itself: in the simulations used in this study, only 32% of annual precipitation in the Amazon has its origin as evapotranspiration within the basin (Staal et al., 2018). This value is consistent with the literature, with most studies reporting between 24% and 40% (Boers et al., 2017; Brubaker et al., 1993; Burde et al., 2006; Costa & Foley, 1999; Eltahir & Bras, 1994; Trenberth, 1999; Tuinenburg et al., 2020; Yang & Dominguez, 2019; Zemp et al., 2014). The difference was caused by the nonlinear relation between atmospheric moisture content and actual precipitation (Neelin et al., 2009). Using an alternative approach to account for this nonlinear relation, we found a 55% reduction of annual precipitation for the 13% loss of atmospheric moisture. Although this was lower than the 70% we found for our main analysis, it still represented a precipitation decrease about four times as large as that under the linearity assumption. The Amazon contribution to rainfall was most important for the west of the study area. The study was based on many simplifying assumptions, which we discuss in detail in the following. Nevertheless, we argue that, given the superlinear relationship between atmospheric moisture content and precipitation was robust, so was the qualitative prediction derived from our results.

The relation between precipitation amount and column water vapor implied a superlinear effect of forest cover on precipitation (Figure 2). This is of particular concern for the effects of the ongoing deforestation, given that its interactions with global climate change and fires may push the Amazon forest across a tipping point, at which self‐amplifying forest loss becomes inevitable (Lovejoy & Nobre, 2018; Zemp, Schleussner, Barbosa, Hirota, et al., 2017). However, our results also implied that increasing forest cover in the Amazon may disproportionally increase precipitation. The extent to which this may occur depended on the distribution of column water vapor. However, the superlinearity led to the general prediction that—given the same initial forest cover—a certain increase in forest cover, via increased transpiration, would enhance precipitation by at least the same amount that an equivalent loss of forest cover would reduce it. Naturally, restoring previously deforested land is expected to compensate for previously lost precipitation due to deforestation, not to overcompensate it. Still, restoring previously deforested lands may benefit drought‐stressed areas downwind from those lands more than is currently assumed. The advantages and disadvantages of forest restoration are currently heavily debated (e.g., Bastin et al., 2019a, 2019b; Lewis et al., 2019; Veldman et al., 2019), but the effects on precipitation have been understudied (Sheil et al., 2019).

Aside from land‐use changes, human activities alter atmospheric moisture content in other ways as well. One effect of the continuing rise in atmospheric CO_2_ concentrations is increased water‐use efficiency of plants: plants will require less water for the same level of photosynthesis. This “CO_2_ fertilization” may reduce plant transpiration and thus decrease atmospheric moisture content (Keenan et al., 2013). If reduced transpiration through land‐use change affects precipitation nonlinearly, then a reduction of transpiration through CO_2_ fertilization would likely have similar effects. Consequently, relatively strong reductions in precipitation levels downwind from forests following CO_2_ fertilization can be hypothesized. Where and under which conditions such effects may be expected is unknown.

The seasonal signal in the reduction of precipitation in relation to moisture loss was less evident than in previous studies (Mu et al., 2021; Staal et al., 2018) because of the nonlinear relationship between column water vapor and precipitation. A small decrease in water vapor flux, as occurring in the wet season, could be enough to lower the column water vapor content to levels at which only small rainfall events can occur (below about 65 mm, see Figure 2), thus lowering the (monthly) precipitation. Indeed, for months with a relatively large decrease in column water vapor, the nonlinear relation between column water vapor and precipitation decreases the differences between the reductions in the dry and wet seasons to the column water vapor. The reductions in precipitation were very high in all seasons; although in an absolute sense the effect may be largest in the wet season, it might be especially relevant in the dry season. We further would like to stress that the quantifications warrant further study, considering the simple approach and the uncertainties involved. Below, we discuss several limitations that are important to account for in future studies, to move from a mainly qualitative result to a robust quantification of the nonlinear effect of column water content loss on precipitation loss in the Amazon and elsewhere.

We used a straightforward approach to address how tree transpiration from the Amazon affects precipitation in the flat parts of the Amazon and close surroundings. Although we moved one step beyond commonly used approaches to estimate transpiration contributions to precipitation, by linking detailed moisture tracking results to empirical patterns in high‐resolution atmospheric reanalysis data, uncertainties and limitations remain. A potential limitation is that, in reality, column water vapor decreases after a precipitation event. Without transpired moisture from the Amazon present in the atmosphere, some events would not occur, and, consequently, neither would the reduction in column water content. In our hypothetical time series without transpiration, we did not account for the presumed higher moisture content of the atmosphere in the absence of a precipitation event. This factor might lead to overestimating the effect of reduced transpiration on precipitation. However, atmospheric moisture often does not rain out only once: on re‐evaporating, the same moisture can again contribute to a precipitation event and would then not be lost from the system (Staal et al., 2018; Zemp et al., 2014). To understand which of these two effects might be most relevant in our case, we analyzed the ERA5 time series of column water content and we determined that the atmospheric water vapor did not decrease significantly as a consequence of rainfall events (Appendix S1). This analysis thus indicated that this limitation is probably not major.

Another limitation results from the implicit assumption that forest loss did not affect wind patterns. Regional circulation models (e.g., Alves et al., 2017) predict changes in atmospheric circulation with implications for the precipitation effects of deforestation (Eiras‐Barca et al., 2020; Ruiz‐Vásquez et al., 2020), but the extent of these changes remains an open question (Marengo et al., 2018). Even the scale and pattern of deforestation might strongly influence precipitation (Lawrence & Vandecar, 2015). Furthermore, we did not include any temperature dependence. Air temperature determines the amount of water necessary to saturate the atmosphere, and, thus, ultimately the relationship between water vapor content and precipitation (as observed by Neelin et al., 2009). The evapotranspirative cooling itself also influences air temperature. In our first‐order approximation, we discarded seasonal variations and temperature dependence, given that temperature in our study area is relatively constant throughout the year. It should also be noted that, when focusing on land areas, rather than the ocean, orographic and surface effects may play a role in the relationship between atmospheric water content and precipitation (Neelin et al., 2009). We tried to minimize such effects by concentrating on a relatively flat target area, excluding the Andes. Finally, we might overestimate the impacts because we estimated the Amazon contribution to rainfall without specifically simulating the transpiration due to the vegetation (e.g., degraded open forest, or crops) that would replace the current forest. Conversely, the moisture recycling data by Staal et al. (2018), used in this study, possibly underestimate it, especially if variability in vertical winds is high.

In addition to the above limitations, our analysis contained uncertainties, as illustrated by our interquartile range for precipitation reduction and the fact that our alternative method yielded an estimate outside that range. Nevertheless, the results strongly indicated that current estimates of precipitation effects of land‐cover changes in the Amazon are underestimated, affecting not just the Amazon basin itself but also the surrounding basins such as the La Plata and Orinoco basins (Staal et al., 2018; Zemp et al., 2014). Furthermore, we suspect that they apply at least partially to the wider tropics. Although the southern Amazon may be a hotspot of transpiration‐induced precipitation (Staal et al., 2018), forests enhance precipitation globally, albeit to unknown extents. Given the rapid land‐cover changes across the globe and climate‐heating‐induced expected changes in precipitation patterns, the physics and ecology of forest–precipitation interactions are an important avenue for future research in global change biology.

## CONFLICT OF INTEREST

We declare no conflict of interest.

## AUTHOR CONTRIBUTIONS

MB, AS, and OAT conceived the project; PF performed a first version of the analysis and wrote a report, on which the paper was based. OAT provided data, and MB performed the analyses, with help from OAT. AS wrote the first draft and led the writing process, while MB led the revision process. All authors contributed critically to the draft and gave final approval for publication.

## Supporting information

Supplementary Material

## Data Availability

The data that support the findings of this study are available from the corresponding authors upon reasonable request.
