# Supplementary material for: Effects of land‐use change in the Amazon on precipitation are likely underestimated
Source: Glob Chang Biol. 2021 Aug 17;27(21):5580–7. doi: 10.1111/gcb.15810 (PMC9291838; doi:10.1111/gcb.15810)
Supplement: Supplementary file 1 — Supplementary Material [file GCB-27-5580-s001.pdf]

Effects of land-use change in the Amazon on precipitation are likely underestimated

Running title: Amazon land-use effects on precipitation

Mara Baudena[1,2,*], Obbe A. Tuinenburg[2], Pendula Ferdinand[2] & Arie Staal[2,*]

ORCID IDs:
Mara Baudena: 0000-0002-6873-6466
Obbe A. Tuinenburg: 0000-0001-6895-0094
Arie Staal: 0000-0001-5409-1436

[1] National Research Council of Italy, Institute of Atmospheric Sciences and Climate (CNR-ISAC), Torino, Italy
[2] Copernicus Institute of Sustainable Development, Utrecht University, Utrecht, the Netherlands

* correspondence to:
Mara Baudena: m.baudena@isac.cnr.it
Arie Staal: a.staal@uu.nl

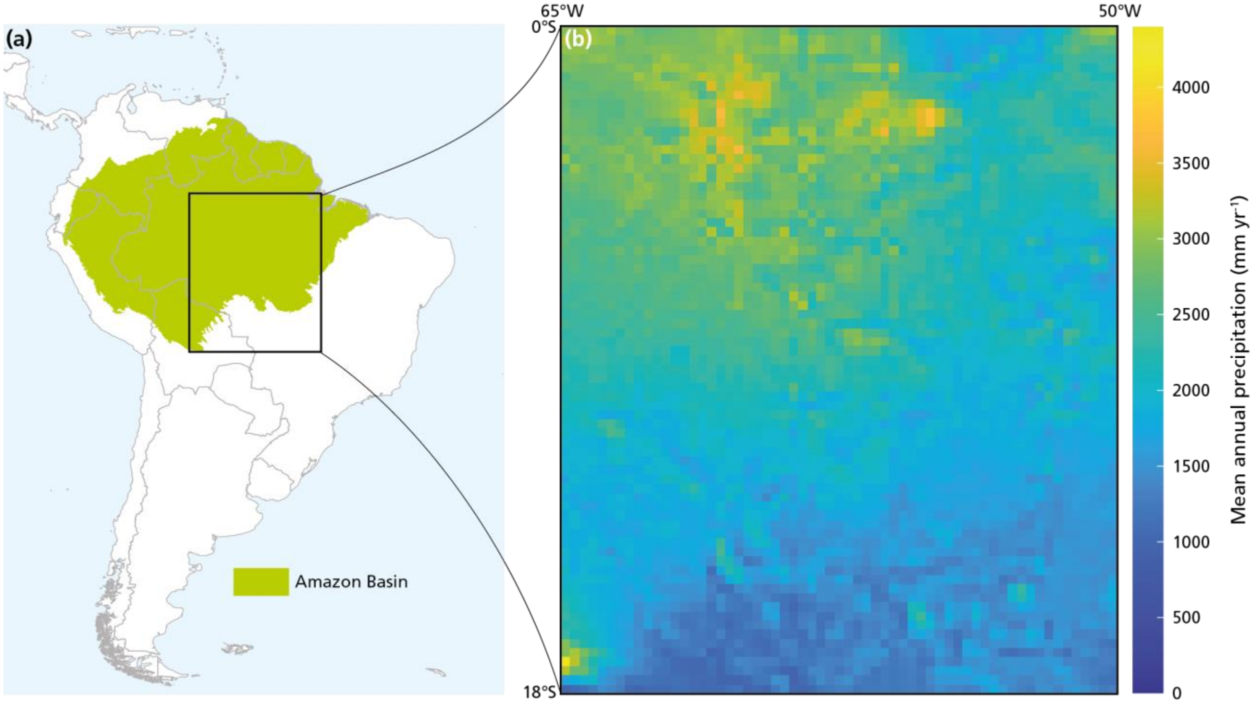

Fig. S1 (a) Map of the Amazon (green shaded area), which is the source of the transpiration for the study area (black rectangle; across 0−18°S and 65−50°W). (b) Map of the mean annual precipitation in the study area (mm yr^{-1}) for the study period (2003−2014).

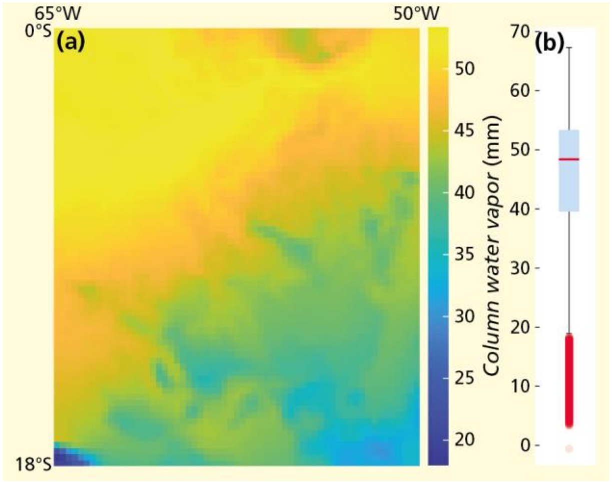

Figure S2: (a) Map of the average column water vapor (*cwv*, mm) in the study period and per each pixel in the study area, and (b) box plot of the daily *cwv* values across the area (mm)

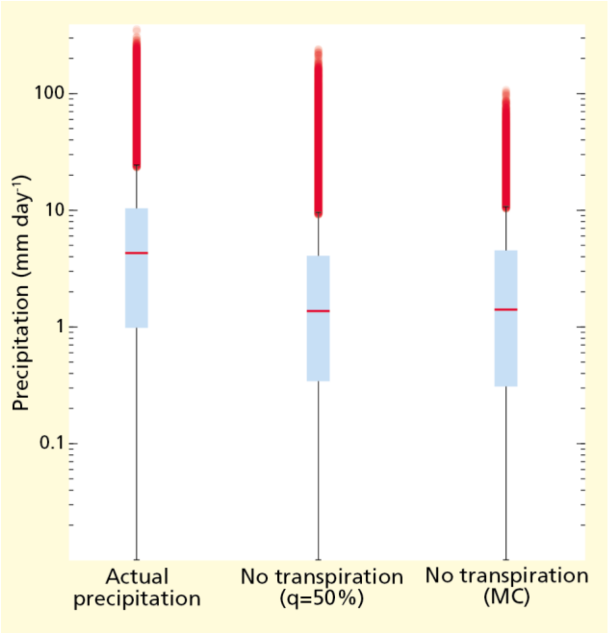

Figure S3: Boxplots of the daily precipitation events (larger than 0.01 mm day^{-1}) across the study area and period for: the actual precipitation (left bar); the simulated precipitation in the absence of the transpiration contribution from the Amazon, as resulting using the median (q=50%, middle bar) and the Monte Carlo approach (MC; right bar). In each box, the central mark indicates the median, and the bottom and top edges of the box indicate the 25th and 75th percentiles, respectively. The whiskers extend to the most extreme data points not considered outliers, and the outliers are plotted individually as red dots.

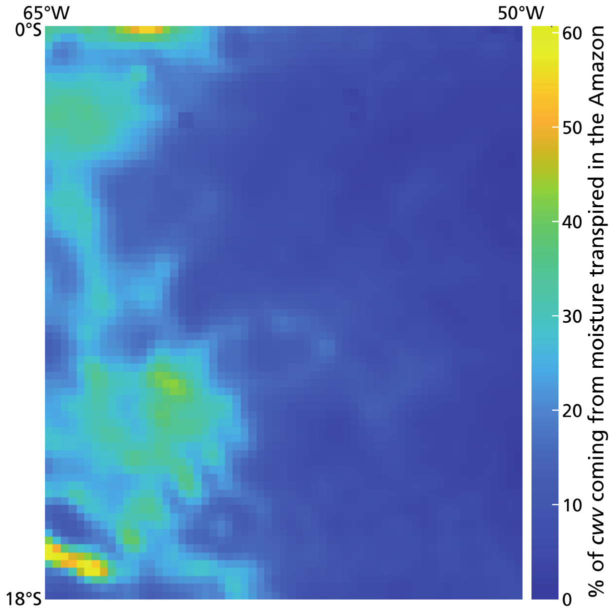

Figure S4 Map of the percentage of column water vapor, *cwv*, in the study area that originates from the Amazon, averaged per each pixel over the period 2003-2014.

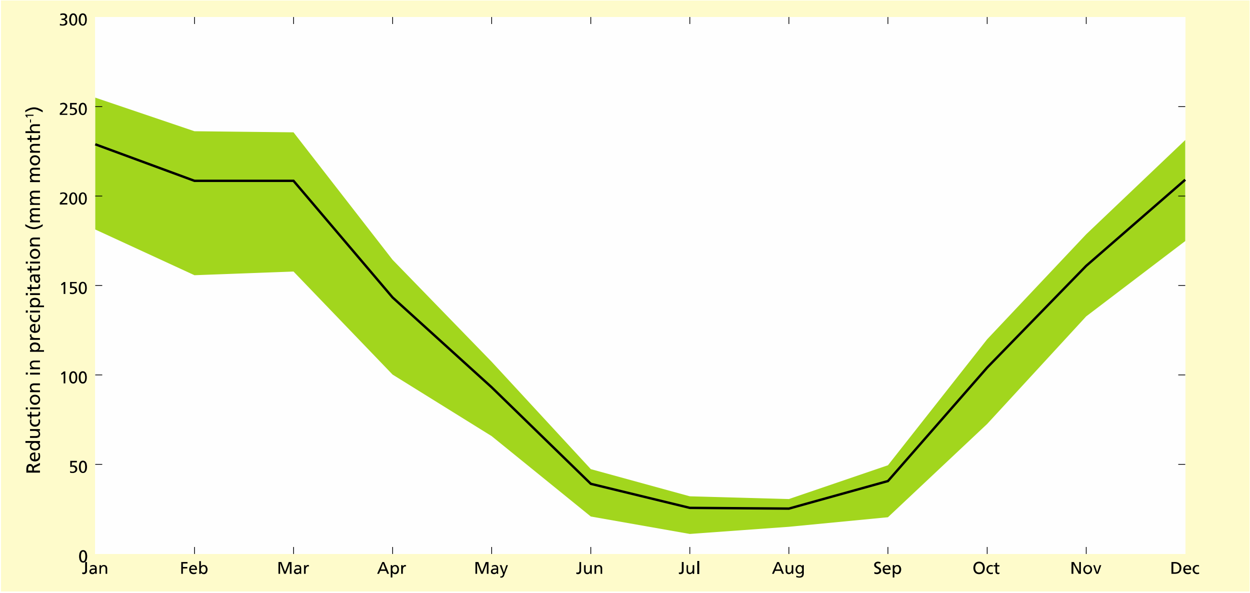

Figure S5: Monthly absolute reduction in precipitation in the study area for 2003–2014 due to the removal of the contribution due to transpiration in the Amazon basin. The black line gives the median ($p_{t,50}$) and the shaded area the interquartile range (between $p_{t,25}$–$p_{t,75}$).

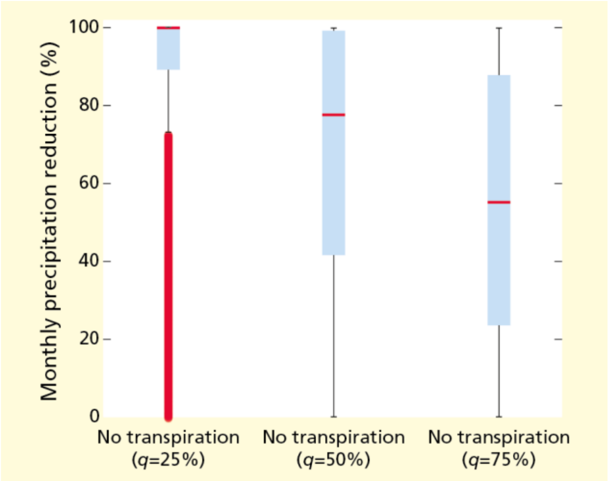

Figure S6: Boxplots of the monthly reduction in precipitation in the absence of transpiration from the Amazon across the study area for 2003−2014, for the lower (*q*=25%), middle (*q*=50%) and higher (*q*=75%) quartiles. In each box, the central red line indicates the median, and the bottom and top edges of the box indicate the 25th and 75th percentiles, respectively. The whiskers extend to the most extreme data points not considered outliers, and the outliers are plotted individually as red dots. Note that in the boxplot of the 25% quantile, the median (99.9 %, red line) is very much shifted towards the high values of the distribution.

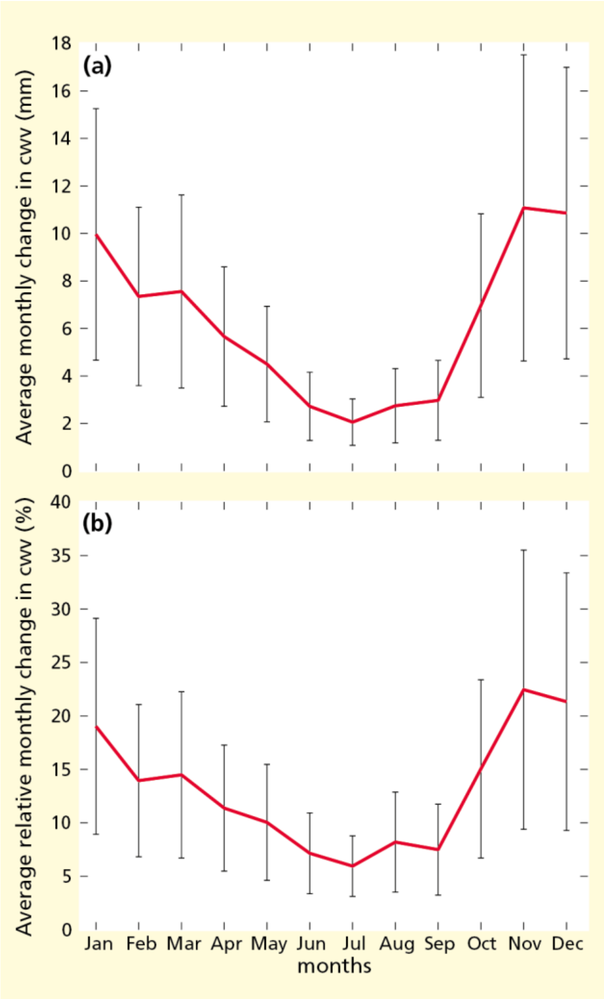

Figure S7. Average monthly change in column water vapor *cwv*, expressed in mm (a) and in percent of the monthly average column water vapor value (b). Monthly averages calculated across the study area and for the period 2003-2014. Error bars indicate the interquartile range.

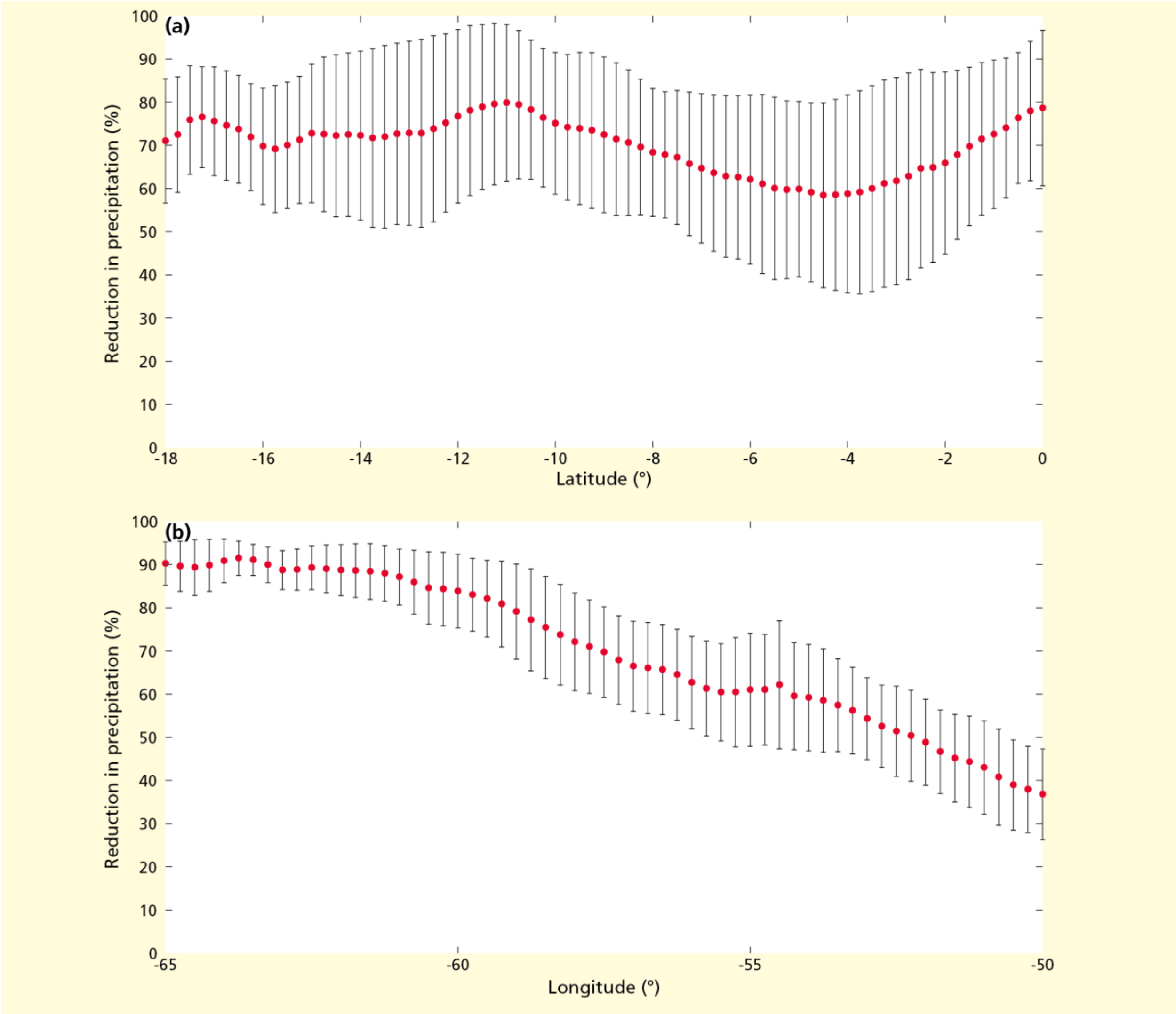

Figure S8: Average percent reduction in yearly precipitation in the study area, as a function of (a) latitude and (b) longitude. Data are averaged every 0.25º across all pixels along the longitude (a) or latitude (b). Error bars indicate one standard deviation.

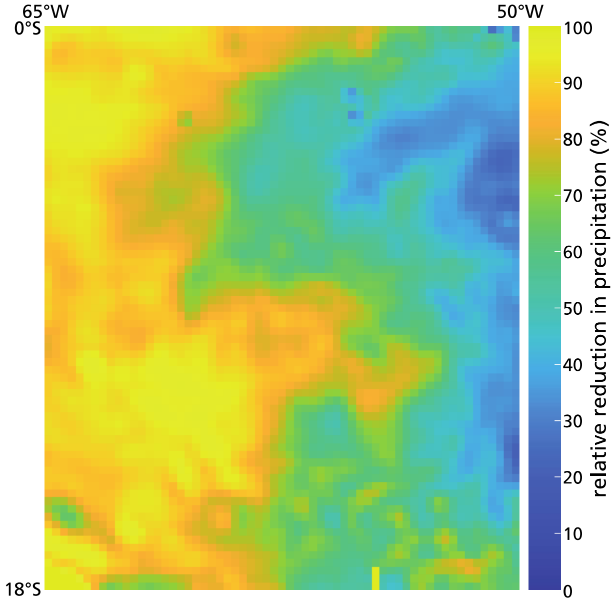

Figure S9: Relative reduction in annual precipitation in the study area (%) in case of absence of transpiration from the Amazon (calculated from the median values $p_{t,50}$).

Appendix 1

How much is column water vapor content *cwv* reduced following a precipitation event?

We looked at the difference between the column water content *cwv* of rainy hours (cwv_{rain}) and of the subsequent hour (cwv_{after}). We identified the rainy hours by using different values as a threshold (between 1 and 100 mm d^{-1}). We calculated the difference cwv_{rain} - cwv_{after} (mm), and the normalized difference, by dividing the former by cwv_{rain}. In the figure S10 here below, we show the distribution of these two variables with box plots (in which a certain hour was identified as rainy if precipitation was larger than 1 mm d^{-1}). Despite the many outliers, the distributions are in fact rather narrow around zero (see median in the Table S1 below).
For all the threshold we used to identify rainy hours (10, 50 and 100 mm d^{-1}), the *cwv* hourly differences are rather close to zero, and the differences below 1% (median value, Table S1 below).
These results indicate that *cwv* seemingly does not reduce much as a consequence of precipitation events. We repeated these analyses also at daily scale, with similar results (not shown).

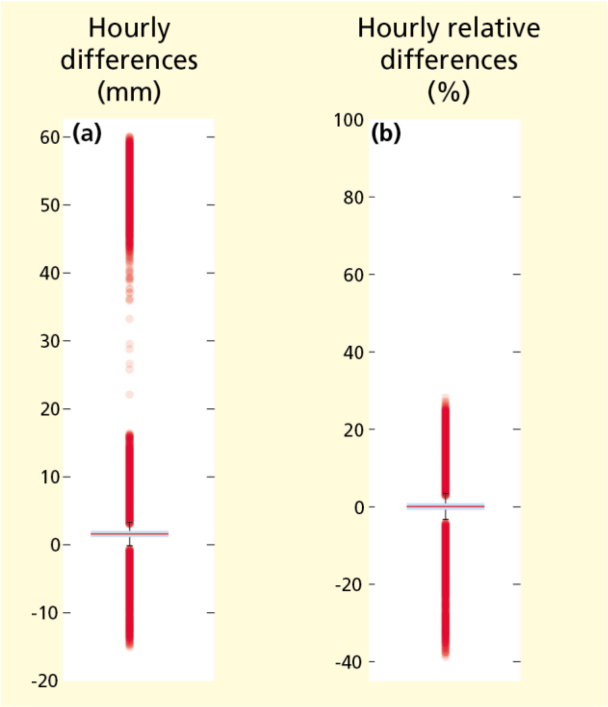

Fig. S10 Boxplots of (a) the differences cwv_{rain}- cwv_{after} (mm), and (b) the normalized differences (cwv_{rain}-cwv_{after})/cwv_{rain} (%). Rainy hours were identified as such if precipitation was larger than 1 mm d^{-1}. In each box, the central mark indicates the median, and the bottom and top edges of the box indicate the 25th and 75th percentiles, respectively. The whiskers extend to the most extreme data points not considered outliers, and the outliers are plotted individually as red dots.

Table S1 Median values of the distribution of the cwv_{rain}- cwv_{after} (mm) and of the normalized differences (cwv_{rain}-cwv_{after})/cwv_{rain}. Rainy hours were identified as such if precipitation rate was larger than the threshold value indicated in the left column. The percentages of total volume of rain represented by such events are reported in the second column.

		**median**	
**threshold p>…**	**% total rain**	**cwv_{rain}-cwv_{after} (mm)**	**normalized difference (%)**
1 mm d^{-1}	99	0.03	0.06
10 mm d^{-1}	84.5	0.13	0.23
50 mm d^{-1}	42	0.24	0.43
100 mm d^{-1}	16	0.32	0.6
